# Maternal and household risk factors for malaria in pregnancy and low birthweight: a prospective cohort study from Uganda

**DOI:** 10.21203/rs.3.rs-7514414/v1

**Published:** 2025-09-17

**Authors:** Miriam Aguti, Jimmy Kizza, Abel Kakuru, Miriam Nakalembe, Joaniter I. Nankabirwa, Stephanie L. Gaw, Bishop Opira, Timothy Ssemukuye, Nida Ozarslan, Anju Ranjit, Erin Cruz, Tamara D. Clark, Michelle E. Roh, Prasanna Jagannathan, Philip J. Rosenthal, Harriet Adrama, Peter Olwoch, Joseph Mayende, Baker Odongo, Ategeka John, Moses R. Kamya, Grant Dorsey

**Affiliations:** Infectious Diseases Research Collaboration; Infectious Diseases Research Collaboration; Infectious Diseases Research Collaboration; Makerere University; Infectious Diseases Research Collaboration; University of California; Infectious Diseases Research Collaboration; Infectious Diseases Research Collaboration; University of California; University of California; University of California; University of California; University of California; Stanford University; University of California; Infectious Diseases Research Collaboration; Infectious Diseases Research Collaboration; Infectious Diseases Research Collaboration; Infectious Diseases Research Collaboration; Infectious Diseases Research Collaboration; Infectious Diseases Research Collaboration; University of California

**Keywords:** Malaria in pregnancy, risk factors, malaria parasitaemia, high grade placental malaria, low-birth weight

## Abstract

**Background:**

Malaria is a leading cause of illness and death in pregnant women and newborns. In 2023, an estimated 12.4 million pregnant women were infected with malaria parasites, resulting in 351,000 low birth weight deliveries. Maternal and household factors associated with malaria in pregnancy and low birth weight were investigated in a high-transmission area of Uganda.

**Methods.:**

Data come from a randomized controlled trial of intermittent preventive treatment in pregnancy (IPTp) conducted from December 2020 to July 2024 in Busia District. Maternal and household data were collected using structured questionnaires. Women were followed through delivery including monthly assessment of microscopic parasitemia, assessment of placental malaria by histopathology, and birth weight. Associations between maternal and household factors were assessed: 1) parasitaemia at enrolment, 2) parasitaemia during pregnancy after starting IPTp, 3) high-grade placental malaria, and 4) low birth weight (< 2500gm).

**Results.:**

Of 2,757 women enrolled, 2,728 (98.9%) had a household survey completed and were included in study. Overall, 38.1% had parasitemia at enrolment, 6.5% had parasitemia following initiation of IPTp, 6.4% had high-grade placental malaria, and 6.8% of live births had low birth weight. Parasitemia at enrolment was more common in those 16–21 years of age (RR = 1.62, 95% CI 1.31–1.99 p < 0.001), primigravida (RR = 1.86, 95% CI 1.57–2.21, p < 0.001)), and living in traditional houses (RR-1.17 95% CI 1.06–1.30, p = 0.002). These associations persisted after IPTp initiation: younger age (RR = 1.72, 95% CI 1.22–2.43, p < 0.002), primigravida (RR = 2.40, 95% CI 1.81–3.20, p < 0.001), and traditional housing (RR = 1.30 95% CI 1.01–1.60, p = 0.02). Maternal level of education was not associated with malaria parasitaemia both at enrollment and after initiation of IPTp. Primigravida was strongly associated with high-grade placental malaria (RR = 4.20, 95% CI 2.33–7.59, p < 0.001)) and low birth weight (RR = 2.14, 95% CI 1.18–3.89, p = 0.01). However, there were no significant associations between maternal age, level of education, household wealth, and household construction with high-grade placental malaria or low birthweight.

**Conclusions.:**

In an area of high malaria transmission, young primigravida women and those living in traditionally constructed houses had the greatest risk of malaria parasitemia during pregnancy. Primigravida women also had higher risks of low birth weight and high grade placental malaria.

## BACKGROUND

Malaria remains a leading cause of morbidity and mortality among pregnant women and their newborns in sub-Saharan Africa. Malaria in pregnancy is associated with maternal anemia and adverse birth outcomes including fetal loss, preterm delivery, low birth weight and neonatal death [1]. In 2023, an estimated 12.4 million pregnant women in sub-Saharan Africa were infected with malaria parasites, resulting in 351,000 newborns with low birth weight [2]. Low birth weight is associated with neonatal and early childhood mortality [3, 4]. To reduce the risk of malaria in pregnancy, the World Health Organization recommends the use of long-lasting insecticidal bed nets (LLINs) and intermittent preventive treatment in pregnancy with sulfadoxine-pyrimethamine (IPTp-SP) starting in the second trimester and given at every antenatal visit at least a month apart. However, the effectiveness of IPTp-SP is threatened by widespread antifolate resistance in East and Southern Africa [5]. Trials evaluating alternative IPTp regimens have shown dihydroartemisinin-piperaquine (DP) to be a promising alternative [6, 7]. In a recent trial of 2,757 pregnant women in Uganda, monthly IPTp with DP was associated with a 94% reduction in clinical malaria and a 97% reduction in the risk microscopic parasitemia during pregnancy compared to IPTp with SP, but IPTp with DP was not associated with reduced risk of adverse birth outcomes, including low birth weight [7]. These findings highlight the need for additional interventions for the prevention of malaria in pregnancy.

To help design new interventions for the prevention of malaria in pregnancy beyond IPTp-SP, it is important to assess risk factors for malaria in pregnancy and low birth weight. These risk factors are multifaceted and often intertwined with household dynamics and maternal health [8]. Studies have shown a range of socio-economic, environmental, and behavioral determinants that influence malaria exposure and its health impacts [8, 9]. Few studies have been conducted to identify risk factors of malaria in pregnancy and low birth weight in Uganda. A study conducted in Eastern Uganda exploring household and maternal risk factors of microscopic and sub-microscopic parasitemia during pregnancy and placental malaria at delivery found that lower gravidity, IPTp with SP compared to DP, poverty, and poor house construction were associated with an increased risk of malaria in pregnancy [8]. However, this prior study did not examine risk factors for low birth weight. In the current study, household and maternal risk factors for measures of malaria in pregnancy and low birth weight were assessed in the same area of Eastern Uganda where the prior study was conducted and malaria is highly endemic. Data for the current study come from 2,757 pregnant women enrolled in a randomized controlled trial of IPTp with monthly SP versus DP versus monthly DP + SP followed through delivery [7]and includes a household survey conducted shortly after enrollment.

## METHODS

### Study setting and participants

This study utilized data from a randomized controlled trial of monthly IPTp with SP vs. DP vs. DP+SP (ClinicalTrials.gov number: NCT04336189) [7]. The trial was conducted between December 2020 and July 2024 in Busia District, south-eastern Uganda. Busia is a high malaria endemic district with perennial transmission that is marked by two annual peaks following the rainy seasons. Prior to 2013, vector control in Busia was limited to targeted distribution of LLINs through antenatal care services. Universal distribution campaigns of free LLINs were conducted in Busia District in May 2013, May 2017, December 2020, and October 2023. The first two campaigns utilized standard pyrethroid LLINs and the last two LLINs containing deltamethrin plus piperonyl butoxide (PermaNet 3.0). Indoor residual spraying has never been implemented in Busia District. Participants were eligible for enrolment in the parent study if they were HIV-uninfected, at least 16 years of age with a viable singleton pregnancy between 12 and 20 weeks of gestation, resided in Busia district, agreed to come for all their medical care at a dedicated study clinic, and provided written informed consent.

### Study procedures

At enrolment, women underwent a standardized medical history/physical examination and had a blood smear done for detection of malaria parasites by microscopy. Women were randomized to one of the IPTp arms and study drugs were given every 4 weeks starting at 16 or 20 weeks gestational age through delivery or 40 weeks gestational age. Following enrolment, women were visited at home where a household survey was conducted to collect detailed information about the participant’s household using a structured questionnaire. Women received all their medical care at a dedicated study clinic open every day. Women were provided with an LLIN following the household survey. Routine assessments were conducted every 4 weeks at the study clinic which included a standardized medical history, physical exam, administration of study drugs, and blood collection for detection of malaria parasites by microscopy. Women were encouraged to deliver at Masafu General Hospital adjacent to the study clinic. Women who delivered at home were visited and encouraged to come to the study clinic shortly after delivery. At delivery, a standardized assessment was made including measuring birth weight and collection of placental tissue for the detection of placental malaria by histopathology.

### Laboratory procedures:

Blood samples for thick blood smears were collected at enrolment and at each routine visit until delivery. Blood smears were stained with 2% Giemsa and read by trained microscopists. A blood smear was considered negative if no asexual parasites were detected after examining 100 high power fields. For quality control, all slides were read by a second microscopist and a third reviewer settled any discrepant readings. Placental tissue collected at delivery was assessed for histological evidence of placental malaria including detection of parasites (active infection) and the proportion of high powered fields with malaria pigment detected (past infection) as previously described [7].

### Data management and analysis

Data were collected at the study clinic and at delivery using standardized case record forms entered into Microsoft Access. Household survey data was collected using handheld computers and customized software designed and programmed to include consistency and range checks. Data were analyzed using Stata version 18.0 (StataCorp, College Station, Tx, USA). The following characteristics were examined as risk factors of malaria in pregnancy and LBW: maternal age, gravidity, bednet ownership at enrolment, maternal education, household wealth, house construction, year of observation, and assigned IPTp regimen. Principal component analysis was used to generate a wealth index based on ownership of common household items which was categorized into tertiles. Houses were categorized based on construction materials as modern (having plaster or cement walls, metal or wooden roofs, and/or closed eaves) or traditional (mud and wattle walls, dirt floors, and/or open eaves). Outcomes of interest included: presence of asexual parasites detected by microscopy at enrolment and during monthly visits after initiation of study drugs; high-grade placental malaria defined as the detection of parasites or malaria pigment in ≥ 30% of high powered fields (HPF) by histopathology which has been associated with adverse birth outcomes [10]; and low birth weight (birth weight <2500g) among live births. Associations between risk factors and dichotomous outcomes were made using bivariate and multivariate log-binomial regression models to obtain risk ratios (RRs). Associations between exposure variables of interest and dichotomous outcomes with repeated measures (microscopic parasitemia following initiation of study drugs) were performed using generalized estimating equations with a log-binomial model and robust standard errors. In all analyses, a p-value <0.05 was considered statistically significant.

### Ethical approval

This study was approved by the Makerere University School of Biomedical Sciences Research Ethics Committee (SBS 714), the Uganda National Council for Science and Technology (HS 2746), the Uganda National Drug Authority (CTC 0135/2020), and the University of California, San Francisco Human Research Protection Program (19–29105).

## RESULTS

### Characteristics of participants and their households.

**Figure 1:**
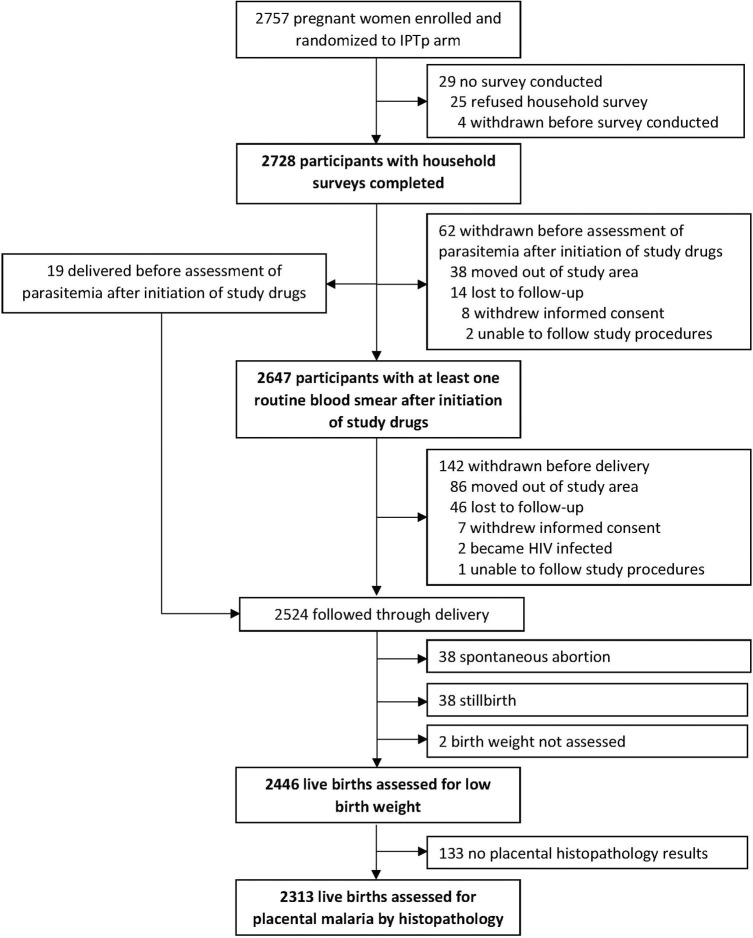
Legend not included with this version.

Enrolment of participants occurred from December 28, 2020 through December 18, 2023. Among 2757 pregnant women enrolled in the parent clinical trial, 2728 (98.9%) had a household survey completed; 25 refused the household survey and 4 were withdrawn before the household survey could be completed (Figure 1). Of the 2728 participants who had a household survey completed, the median age was 23 years (range 16–44), 51.8% were multigravida, 53.9% reported owning a bed net at the time of enrolment, 68.9% had only primary school or no formal education, and 67.0% lived in a house with traditional construction (Table 1). There were no significant differences in characteristics of study participants across the IPTp arms (Table 1). Of the 2728 participants who had household survey completed, 2647 (97.0%) had at least one blood smear after initiation of study drugs, 2524 (92.5%) were followed up to delivery, and 2313 (85.1%) had a live birth with assessment for placental malaria by histopathology (Figure 1).

### Factors associated with microscopic parasitemia at enrolment.

At enrolment, 38.1% of participants had malaria parasites detected by microscopy. Risk factors for parasitemia at enrolment are presented in Table 2. In multivariate analysis, younger age was associated with an increased risk of parasitemia, with women 16–21 years of age having a 62% greater risk of parasitemia compared to women 28–44 years of age (RR= 1.62, 95% CI 1.31–1.99, p<0.001). Similarly, lower gravidity was associated with a higher risk of parasitemia, with primigravida women having an 86% greater risk of parasitemia compared to multigravida women (RR=1.86, 95% CI 1.57–2.21, p<0.001). There were no significant associations between household wealth, bednet ownership at enrolment, and level of education with parasitemia in multivariate analysis. Women who lived in traditional houses had a 17% greater risk of parasitemia compared to those who lived in modern houses (RR=1.17, 95% CI 1.06–1.30, p=0.002). The risk of parasitemia at enrolment increased over the course of the study, with women enrolled in 2023 having a 29% greater risk of parasitemia compared to women enrolled in 2021 (RR=1.29, 95% CI 1.15–1.45, p<0.001).

### Factors associated with microscopic parasitemia following initiation of study drugs.

Following initiation of study drugs, 6.5% of participants had malaria parasites detected by microscopy at the time of routine visits conducted every 4 weeks over the course of the study. Risk factors for parasitemia at the time of routine visits following the initiation of study drugs are presented in Table 3. Similar to parasitemia at enrolment, lower gravidity and younger age were independently associated with an increased risk of parasitemia after initiation of study drugs in multivariate analysis. Women 16–21 years of age had a 72% higher risk of parasitemia compared to women 28–44 years of age (RR=1.72, 95% CI 1.22–2.43, p=0.002). Primigravida women had more than twice the risk of microscopic parasitemia compared to multigravida women (RR=2.40, 95% CI 1.81–3.20, p<0.001). Women living in the poorest households had a 30% higher risk of parasitemia compared to women living in the least poor households (RR=1.30, 95% CI 1.05–1.60, p=0.02). Women who lived in traditional houses had a 28% greater risk of parasitemia compared to those who lived in modern houses (RR=1.28, 95% CI 1.05–1.55, p=0.01). Level of education was not significantly associated with the risk of parasitemia after the initiation of study drugs. The risk of parasitemia during routine assessments increased from 2021 to 2023, then decreased in 2024 after enrolment had been completed. As previously described, women randomized to IPTp regimens containing DP had a much lower risk of parasitemia following the initiation of study drugs, with the DP arm associated with a 97% reduction (RR=0.03, 95% CI 0.02–0.05, p<0.001) and the DP+SP arm associated with a 93% reduction (RR=0.07, 95% CI 0.05–0.10, p<0.001) compared to the SP arm[7].

### Factors associated with high grade placental malaria

There were 2313 women who were assessed for placental malaria by histopathology, of which 1147 (49.6%) had no evidence of placental malaria (no parasites or malaria pigment detected), 1019 (46.6%) had evidence of mild-moderate placental malaria (malaria pigment detected in < 30% of HPF), and 147 (6.4%) had evidence of high-grade placental malaria (detection of parasites or malaria pigment detected in ≥ 30% of HPF). Risk factors for high-grade placental malaria are presented in Table 4. In multivariate analysis, lower gravidity was associated with an increased risk of high-grade placental malaria, with primigravida women having over 4 times greater risk of compared to multigravida women (RR=4.20, 95% CI 2.33–7.59, p<0.001). There were no significant associations between age, household wealth, level of education, or house construction with high-grade placental malaria in multivariate analysis. The risk of high-grade placental malaria increased over the course of the study with women delivering in 2024 having over 4 times the risk of high-grade placental malaria compared to women who delivered in 2021 (RR=4.62, 95% CI 2.22–9.65, p<0.001). As previously described, women randomized to IPTp regimens containing DP had a much lower risk of high-grade placental malaria, with the DP arm associated with an 88% reduction (RR=0.12, 95% CI 0.07–0.20, p<0.001) and the DP+SP arm associated with an 86% reduction (RR=0.14, 95% CI 0.09–0.23, p<0.001) compared to the SP arm[7].

### Factors are associated with low birth weight

Among 2313 live births with placental histopathology assessed, 157 (6.8%) had low birth weight (< 2500 grams). Risk factors for low birth weight are presented in Table 5. In a multivariate analysis, lower gravidity was associated with a higher risk of low birth weight, with primigravida women having over twice the risk of low birth weight compared to multigravida women (RR= 2.14, 95% CI 1.18–3.89, p=0.01). The risk of low birth weight decreased in each subsequent year during the first 3 years of the study (but not the last year), with women who delivered in 2023 having 41% lower risk compared to women who delivered in 2021 (RR=0.59, 95% CI 0.37–0.95, p=0.03). Women with high-grade placental malaria had over 3 times the risk of delivering a low birth weight infant compared to women with no evidence of placental malaria (RR=3.02, 95% CI 1.77–5.16, p<0.001). Interestingly, as previously described, women randomized to IPTp regimens containing DP had a higher risk of delivering a low birth weight infant, with the DP arm associated with a 64% increase (RR=1.64, 95% CI 1 .08–2.50, p=0.01) and the DP+SP arm associated with an 85% increase (RR=1.85, 95% CI 1.24–2.77, p=0.003) compared to the SP arm [7]. Maternal age, household wealth, level of education, or house construction were not significantly associated low birth weight risk in multivariate analysis.

## DISCUSSION

This study utilized data from a large randomized controlled trial of IPTp conducted in a rural, high transmission setting in Uganda to evaluate the risk factors of parasitemia, high-grade placental malaria, and low birth weight. Gravidity was the strongest and most consistent risk factor for malaria and LBW: primigravida women had approximately a two-fold higher risk of parasitemia before and after IPTp initiation, over 4 times the risk of placental malaria, and more than twice the risk of delivering a low-birth-weight infant compared to multigravida women. Independent of gravidity, younger maternal age was associated with a higher risk of parasitemia before and after initiation of IPTp, but not a significant increase in the risk of placental malaria or low birth weight. Living in a traditional house compared to a modern house was associated with a higher risk of parasitemia before and after initiation of IPTp but not placental malaria or low birth weight. Living in the poorest houses was only significantly associated with parasitemia following initiation of IPTp. Gravidity was the strongest risk factor for malaria and low birth weight, with primigravida women at a significantly higher risk than multigravidas, while younger age and living in a traditionally constructed house were also associated with increased risk of parasitemia.

In high transmission settings, lower gravidity has consistently been identified as a key risk factor for malaria in pregnancy [11–13]. This study reaffirmed the phenomenon of “gravidity dependent immunity”, with primigravida women at highest risk of infection with malaria parasites both before and after receiving IPTp [14]. Although maternal age and gravidity are highly correlated, younger age has also been associated with an increased risk of infection with malaria parasites independent of gravidity [15], as observed in this study. Indeed, in this study the risk of parasitemia at enrollment was 65.1% among primigravida women 21 years or younger compared to 20.5% among multigravida women 28 years or older. These findings highlight the importance of targeting young, primigravida women for malaria prevention measures at the time of their first antenatal visit. Lower gravidity was also a strong risk factor for high-grade placental malaria, with primigravid women having a four-fold increased risk compared to multigravida women. Although in bivariate analyses, younger women had a 5-fold higher risk of high-grade placental malaria, this association was attenuated and not statistically significant in multivariable analyses, suggesting that much of this effect was potentially mediated by other risk factors. Notably, the presence of high-grade placental malaria was strongly associated with low birth weight, consistent with previous findings [14, 16]. A distinctive aspect of this study was the assessment of risk factors associated with delivering low birth weight infants; primigravidae had over twice the risk of low birth weight compared to multigravidae, consistent with previous studies [17–20]. In contrast, maternal age was not significantly associated with low birth weight independent of gravidity. Prior studies have reported mixed findings, with some studies reporting maternal age under 25 years [17–20] and over 35 years [21] associated with an increased risk of low birth weight, while others found no significant association between maternal age and low birth weight [21].

Considering household and other maternal demographic factors, living in a house constructed of traditional materials versus a modern house was associated with an increased risk of malaria parasitemia. Indeed, poor quality housing is a well-documented risk factor for infection with malaria parasites and has become a target for new malaria control interventions aimed at improving housing quality [22]. In contrast, house construction was not independently associated with high-grade placental malaria or low birth weight. With the exception of women from the poorest households being associated with an increased risk of parasitemia following initiation of study drugs, household wealth and maternal education were not significant, independent risk factors for any of the outcomes in this study. In contrast, evidence from previous studies has shown associations between improved socio-economic status and higher levels of education with better implementation of appropriate malaria control interventions and a lower burden of malaria in non-pregnant populations [23, 24].

Strengths of this study include a large sample size nested within a randomized controlled trial conducted over several years. However, this study also had several limitations. Firstly, given the observational nature of the risk factor analysis, estimated associations may have been biased due to unmeasured confounders. Secondly, our study was conducted in an area of high malaria transmission intensity and widespread SP resistance; findings may not be generalizable to regions where SP resistance is not widespread or where malaria transmission is low to moderate. Third, as this was a secondary analysis of data from a randomized controlled trial, it was not possible to evaluate the full spectrum of risk factors for malaria in pregnancy, high grade placental malaria, and low birth weight. Finally, with the exception of IPTp regimens (which were randomly assigned), associations between risk factors and outcomes are not evidence of causality. For example, malaria may contribute to poverty, limit educational attainment, and influence the choice of housing materials through its direct and indirect socioeconomic impacts.

## CONCLUSION

In this study from a high transmission area of Uganda, primigravida women were at highest risk of measures of malaria in pregnancy and the delivery of low birth weight infants. Younger maternal age, poor house construction, and lower socioeconomic status were also associated with an increased risk of parasitemia during pregnancy. The risks of malaria in pregnancy and low birth weight varied from year to year, following different patterns, suggesting the presence of temporally varying unmeasured risk factors. Therefore, we suggest that interventions which target young and primigravida mothers and involve improvement of housing types should be introduced in order to reduce the burden on malaria in pregnancy.

## Figures and Tables

**Table 1.** Characteristics of study participants at enrollment stratified by IPTp am

Variable	Categories	IPTp am			
		All (n=2728)	SP (n=908)	DP (n=909	DP+SP (n=911)
Age in years	28–44	846 (31.0%)	288 (31.7%)	286 (31.5%)	272 (29.9%)
	22–27	838 (30.7%)	279 (30.7%)	287 (31.6%)	272 (29.9%)
	16–21	1044 (38.3%)	341 (37.6%)	336 (37.0%)	367 (40.3%)
Gravidity	Multigravida	1414 (51.8%)	480 (52.9%)	477 (52.5%)	457 (50.2%)
	Secundigravida	598 (21.9%)	201 (22.1%)	202 (22.2%)	195 (21.4%)
	Primigravida	716 (26.3%)	227 (25.0%)	230 (25.3%)	259 (28.4%)
Wealth index	Least poor	906 (33.2%)	290 (31.9%)	301 (33.1%)	315 (34.6%)
	Middle	912 (33.4%)	307 (33.8%)	305 (33.6%)	300 (32.9%)
	Poorest	910 (33.4%)	311 (34.3%)	303 (33.3%)	296 (32.5%)
Bednet ownership	Yes	1470 (53.9%)	491 (54.1%)	480 (52.8%)	499 (54.8%)
	No	1258 (46.1%)	417 (45.9%)	429 (47.2%)	412 (45.2%)
Highest level of education	Secondary or higher	848 (31.1%)	279 (30.7%)	290 (31.9%)	279 (30.6%)
	Primary or none	1880 (68.9%)	629 (69.3%)	619 (68.1%)	632 (69.4%)
Type of house construction	Modern	901 (33.0%)	309 (34.0%)	286 (31.5%)	306 (33.6%)
	Traditional	1827 (67.0%)	599 (66.0%)	623 (68.5%)	605 (66.4%)
Year of enrollment	2021*	731 (26.8%)	243 (26.8%)	244 (26.8%)	244 (26.8%)
	2022	984 (36.1%)	328 (36.1%)	326 (35.9%)	330 (36.2%)
	2023	1013 (37.1%)	337 (37.1%)	339 (37.3%)	337 (37.0%)

* includes 19 participants enrolled December 28–31, 2020

**Table 2.** Factors associated with microscopic parasitemia at enrollment

Variable	Categories	Parasitemia, n/N (%)	Univariate analysis		Multivariate analysis	
			RR (95% CI)	p-value	RR (95% CI)	p-value
Age in years	28–44	176/846 (20.8%)	Reference		Reference	
	22–27	275/838 (32.8%)	1.58 (1.34–1.86)	<0.001	1.36 (1.14–1.62)	0.001
	16–21	588/1044 (56.3%)	2.71 (2.35–3.12)	<0.001	1.62 (1.31–1.99)	<0.001
Gravidity	Multigravida	346/1414 (24.5%)	Reference		Reference	
	Secundigravida	239/598 (40.0%)	1.63 (1.43–1.87)	<0.001	1.26 (1.07–1.49)	0.006
	Primigravida	454/716 (63.4%)	2.59 (2.33–2.88)	<0.001	1.86 (1.57–2.21)	<0.001
Wealth index	Least poor	302/906 (33.3%)	Reference		Reference	
	Middle	339/912 (37.2%)	1.12 (0.98–1.26)	0.09	1.06 (0.95–1.19)	0.29
	Poorest	398/910 (43.7%)	1.31 (1.17–1.48)	<0.001	1.08 (0.96–1.20)	0.19
Bednet ownership	Yes	486/1470 (33.1%)	Reference		Reference	
	No	553/1258 (44.0%)	1.33 (1.21–1.46)	<0.001	1.07 (0.98–1.17)	0.13
Highest level of education	Secondary or higher	303/848 (35.7%)	Reference		Reference	
	Primary or none	736/1880 (39.2%)	1.10 (0.99–1.22)	0.09	1.10 (1.00–1.21)	0.06
Type of house construction	Modern	283/901 (31.4%)	Reference		Reference	
	Traditional	756/1827 (41.4%)	1.32 (1.18–1.47)	<0.001	1.17 (1.06–1.30)	0.002
Year of enrollment	2021*	228/731 (31.2%)	Reference		Reference	
	2022	374/984 (38.0%)	1.22 (1.07–1.39)	0.004	1.23 (1.09–1.38)	0.001
	2023	437/1013 (43.1%)	1.38 (1.22–1.57)	<0.001	1.29 (1.15–1.45)	<0.001

* includes 19 participants enrolled December 28–31, 2020

**Table 3.** Factors associated with microscopic parasitemia following initiation of study drugs

Variable	Categories	Parasitemia, n/N (%)	Univariate analysis		Multivariate analysis	
			RR (95% CI)	p-value	RR (95% CI)	p-value
Age in years	28–44	133/4230 (3.1%)	Reference		Reference	
	22–27	209/4006 (5.2%)	1.66 (1.24–2.29)	0.001	1.38 (1.04–1.83)	0.03
	16–21	504/4753 (10.6%)	3.45 (2.66–4.48)	<0.001	1.72 (1.22–2.43)	0.002
Gravidity	Multigravida	260/6984 (3.7%)	Reference		Reference	
	Secundigravida	201/2798 (7.2%)	1.89 (1.47–2.44)	<0.001	1.44 (1.08–1.91)	0.01
	Primigravida	385/3207 (12.0%)	3.29 (2.64–4.10)	<0.001	2.40 (1.81–3.20)	<0.001
Wealth index	Least poor	203/4328 (4.7%)	Reference		Reference	
	Middle	276/4422 (6.2%)	1.32 (1.01–1.72)	0.04	1.14 (0.91–1.43)	0.24
	Poorest	367/4239 (8.7%)	1.84 (1.44–2.36)	<0.001	1.30 (1.05–1.60)	0.02
Highest level of education	Secondary or higher	213/4022 (5.3%)	Reference		Reference	
	Primary or none	633/8967 (7.1%)	1.34 (1.07–1.67)	0.01	1.16 (0.96–1.40)	0.14
Type of house construction	Modern	214/4275 (5.0%)	Reference		Reference	
	Traditional	632/8714 (7.3%)	1.45 (1.15–1.83)	0.002	1.28 (1.05–1.55)	0.01
Year of assessment	2021	101/2258 (4.5%)	Reference		Reference	
	2022	326/4807 (6.8%)	1.35 (1.01–1.82)	0.05	1.47 (1.13–1.92)	0.004
	2023	355/4671 (7.6%)	1.44 (1.07–1.94)	0.02	1.64 (1.26–2.13)	<0.001
	2024	64/1253 (5.1%)	1.00 (0.66–1.50)	0.99	1.17 (0.80–1.70)	0.42
IPTp arm	SP	764/4324 (17.7%)	Reference		Reference	
	DP	23/4326 (0.5%)	0.03 (0.02–0.05)	<0.001	0.03 (0.02–0.05)	<0.001
	DP+SP	59/4339 (1.4%)	0.08 (0.06–0.10)	<0.001	0.07 (0.05–0.10)	<0.001

**Table 4.** Factors associated with high-grade placental malaria

Variable	Categories	Placental malaria, n/N (%)	Univariate analysis		Multivariate analysis	
			RR (95% CI)	p-value	RR (95% CI)	p-value
Age in years	28–44	17/751 (2.3%)	Reference		Reference	
	22–27	28/710 (3.9%)	1.74 (0.96–3.15)	0.07	1.23 (0.65–2.34)	0.53
	16–21	102/852 (12.0%)	5.29 (3.20–8.75)	<0.001	1.83 (0.89–3.75)	0.10
Gravidity	Multigravida	31/1238 (2.5%)	Reference		Reference	
	Secundigravida	29/498 (5.8%)	2.33 (1.42–3.82)	0.001	1.73 (0.95–3.15)	0.08
	Primigravida	87/577 (15.1%)	6.02 (4.04–8.96)	<0.001	4.20 (2.33–7.59)	<0.001
Wealth index	Least poor	40/770 (5.2%)	Reference		Reference	
	Middle	47/790 (6.0%)	1.15 (0.76–1.73)	0.52	0.86 (0.60–1.22)	0.40
	Poorest	60/753 (8.0%)	1.53 (1.04–2.26)	0.03	0.97 (0.69–1.36)	0.86
Highest level of education	Secondary or higher	33/711 (4.6%)	Reference		Reference	
	Primary or none	114/1602 (7.1%)	1.53 (1.05–2.24)	0.03	1.36 (0.97–1.91)	0.08
Type of house construction	Modern	39/757 (5.2%)	Reference		Reference	
	Traditional	108/1556 (6.9%)	1.35 (0.94–1.92)	0.10	1.13 (0.83–1.53)	0.43
Year of delivery	2021	7/309 (2.3%)	Reference		Reference	
	2022	35/748 (4.7%)	2.07 (0.93–4.60)	0.08	2.00 (0.93–4.29)	0.08
	2023	63/841 (7.5%)	3.31 (1.53–7.14)	0.002	3.34 (1.61–6.94)	0.001
	2024	42/415 (10.1%)	4.47 (2.03–9.81)	<0.001	4.62 (2.22–9.65)	<0.001
IPTp arm	SP	116/762 (15.2%)	Reference		Reference	
	DP	14/776 (1.8%)	0.12 (0.07–0.20)	<0.001	0.12 (0.07–0.20)	<0.001
	DP+SP	17/775 (2.2%)	0.14 (0.09–0.24)	<0.001	0.14 (0.09–0.23)	<0.001

**Table 5.** Factors associated with low birth weight

Variable	Categories	Low birth weight n/N (%)	Univariate analysis		Multivariate analysis	
			RR (95% CI)	p-value	RR (95% CI)	p-value
Age in years	28–44	41/751 (5.5%)	Reference		Reference	
	22–27	38/710 (5.4%)	0.98 (0.64–1.51)	0.93	0.81 (0.50–1.31)	0.39
	16–21	78/852 (9.2%)	1.68 (1.16–2.42)	0.006	0.77 (0.41–1.44)	0.42
Gravidity	Multigravida	62/1238 (5.0%)	Reference		Reference	
	Secundigravida	32/498 (6.4%)	1.28 (0.85–1.94)	0.24	1.45 (0.84–2.49)	0.18
	Primigravida	63/577 (10.9%)	2.18 (1.56–3.05)	<0.001	2.14 (1.18–3.89)	0.01
Wealth index	Least poor	53/770 (6.9%)	Reference		Reference	
	Middle	49/790 (6.2%)	0.90 (0.62–1.31)	0.59	0.88 (0.60–1.28)	0.50
	Poorest	55/753 (7.3%)	1.06 (0.74–1.53)	0.75	0.93 (0.64–1.37)	0.73
Highest level of education	Secondary or higher	39/711 (5.5%)	Reference		Reference	
	Primary or none	118/1602 (7.4%)	1.34 (0.95–1.91)	0.10	1.41 (0.98–2.02)	0.06
Type of house construction	Modern	50/757 (6.6%)	Reference		Reference	
	Traditional	107/1556 (6.9%)	1.04 (0.75–1.44)	0.81	1.00 (0.71–1.41)	0.99
Year of delivery	2021	26/309 (8.4%)	Reference		Reference	
	2022	56/748 (7.5%)	0.89 (0.57–1.39)	0.61	0.83 (0.53–1.29)	0.41
	2023	47/841 (5.6%)	0.66 (0.42–1.05)	0.08	0.59 (0.37–0.95)	0.03
	2024	28/415 (6.8%)	0.80 (0.48–1.34)	0.40	0.68 (0.40–1.14)	0.14
Placental malaria categories	No parasites or pigment	66/1147 (5.8%)	Reference		Reference	
	No parasites, pigment < 30% HPF	67/1019 (6.6%)	1.14 (0.82–1.59)	0.43	1.03 (0.72–1.50)	0.84
	Parasites or pigment ≥ 30% HPF	24/147 (16.3%)	2.84 (1.84–4.38)	<0.001	3.02 (1.77–5.16)	<0.001
IPTp arm	SP	41/762 (5.4%)	Reference		Reference	
	DP	53/776 (6.8%)	1.27 (0.85–1.88)	0.24	1.64 (1.08–2.50)	0.02
	DP+SP	63/775 (8.1%)	1.51 (1.03–2.21)	0.03	1.85 (1.24–2.77)	0.003

## Data Availability

The datasets reported herein will be made available from the corresponding author on reasonable request.

## Abbreviations

DP: Dihydroartemisinin-piperaquine
SP: Sulfadoxine–pyrimethamine
IPTp: Intermittent preventive treatment for malaria during pregnancy
LLIN: Long Lasting Insecticidal Net
MOH: Ministry of Health
qPCR: Quantitative polymerase chain reaction
HPF: High Powered Fields
UCSF: University of California San Francisco
WHO: World Health Organization
IDRC: Infectious Diseases Research Collaboration

## References

[1] Satapathy P, Khatib MN, Gaidhane S, Zahiruddin QS, Sharma RK, Rustagi S, Al-Jishi JM, Albayat H, Al Fares MA, Garout M, Adverse pregnancy outcomes in maternal malarial infection: A systematic review and meta-analysis. New Microbes New Infect. 2024;62:101474. 39286328 10.1016/j.nmni.2024.101474 PMC11403273

[2] Venkatesan P. WHO world malaria report 2024. Lancet Microbe 2025:101073. 39923782 10.1016/j.lanmic.2025.101073

[3] Chang YS, Liang FW, Lin YJ, Lu TH, Lin CH. Neonatal and infant mortality of very-low-birth-weight infants in Taiwan: Does the level of delivery hospital matter? Pediatr Neonatol. 2021;62:419–27. 34020899 10.1016/j.pedneo.2021.04.003

[4] McCormick MC. The contribution of low birth weight to infant mortality and childhood morbidity. N Engl J Med. 1985;312:82–90. 3880598 10.1056/NEJM198501103120204

[5] Sundararaman SA, Odom John AR. Prevention of malaria in pregnancy: The threat of sulfadoxine-pyrimethamine resistance. Front Pediatr. 2022;10:966402. 36061376 10.3389/fped.2022.966402 PMC9433640

[6] Kakuru A, Jagannathan P, Muhindo MK, Natureeba P, Awori P, Nakalembe M, Opira B, Olwoch P, Ategeka J, Nayebare P, Dihydroartemisinin-Piperaquine for the Prevention of Malaria in Pregnancy. N Engl J Med. 2016;374:928–39. 26962728 10.1056/NEJMoa1509150 PMC4847718

[7] Kakuru A, Kizza J, Aguti M, Adrama H, Ategeka J, Olwoch P, Nakalembe M, Nankabirwa JI, Opira B, Ozarslan N [Preprint] Dihydroartemisinin–piperaquine plus sulfadoxine-pyrimethamine versus either drug alone for intermittent preventive treatment of malaria in pregnancy: a double-blind, randomized, controlled trial. medRxiv 2025:2025.2003.2024.25324508.. 10.1371/journal.pmed.1004582 PMC12445490 40966190

[8] Okiring J, Olwoch P, Kakuru A, Okou J, Ochokoru H, Ochieng TA, Kajubi R, Kamya MR, Dorsey G, Tusting LS. Household and maternal risk factors for malaria in pregnancy in a highly endemic area of Uganda: a prospective cohort study. Malar J. 2019;18:144. 31014336 10.1186/s12936-019-2779-x PMC6480498

[9] Tapias-Rivera J, Gutiérrez JD. Environmental and socio-economic determinants of the occurrence of malaria clusters in Colombia. Acta Trop. 2023;241:106892. 36935051 10.1016/j.actatropica.2023.106892

[10] Ategeka J, Kakuru A, Kajubi R, Wasswa R, Ochokoru H, Arinaitwe E, Yeka A, Jagannathan P, Kamya MR, Muehlenbachs A, Relationships Between Measures of Malaria at Delivery and Adverse Birth Outcomes in a High-Transmission Area of Uganda. J Infect Dis. 2020;222:863–70. 32249917 10.1093/infdis/jiaa156 PMC7399701

[11] Alruwaili M, Uwimana A, Sethi R, Murindahabi M, Pierce field E, Umulisa N, Abram A, Eckert E, Munguti K, Mbituyumuremyi A, Peripheral and Placental Prevalence of Sulfadoxine-Pyrimethamine Resistance Markers in Plasmodium falciparum among Pregnant Women in Southern Province, Rwanda. Am J Trop Med Hyg. 2023;109:1057–62. 37783456 10.4269/ajtmh.23-0225 PMC10622487

[12] Nnaji GA, Okafor CI, Ikechebelu JI. An evaluation of the effect of parity and age on malaria parasitaemia in pregnancy. J Obstet Gynaecol. 2006;26:755–8. 17130024 10.1080/01443610600956089

[13] Nosten F, ter Kuile F, Maelankirri L, Decludt B, White NJ. Malaria during pregnancy in an area of unstable endemicity. Trans R Soc Trop Med Hyg. 1991;85:424–9. 1836685 10.1016/0035-9203(91)90205-d

[14] Bihoun B, Zango SH, Traoré-Coulibaly M, Valea I, Ravinetto R, Van Geertruyden JP, D'Alessandro U, Tinto H, Robert A. Age-modified factors associated with placental malaria in rural Burkina Faso. BMC Pregnancy Childbirth. 2022;22:248. 35331181 10.1186/s12884-022-04568-4 PMC8951713

[15] Desai M, ter Kuile FO, Nosten F, McGready R, Asamoa K, Brabin B, Newman RD. Epidemiology and burden of malaria in pregnancy. Lancet Infect Dis. 2007;7:93–104. 17251080 10.1016/S1473-3099(07)70021-X

[16] Tran EE, Cheeks ML, Kakuru A, Muhindo MK, Natureeba P, Nakalembe M, Ategeka J, Nayebare P, Kamya M, Havlir D, The impact of gravidity, symptomatology and timing of infection on placental malaria. Malar J. 2020;19:227. 32580739 10.1186/s12936-020-03297-3 PMC7315526

[17] Demelash H, Motbainor A, Nigatu D, Gashaw K, Melese A. Risk factors for low birth weight in Bale zone hospitals, South-East Ethiopia: a case-control study. BMC Pregnancy Childbirth. 2015;15:264. 26463177 10.1186/s12884-015-0677-y PMC4604703

[18] Momeni M, Danaei M, Kermani AJ, Bakhshandeh M, Foroodnia S, Mahmoudabadi Z, Amirzadeh R, Safizadeh H. Prevalence and Risk Factors of Low Birth Weight in the Southeast of Iran. Int J Prev Med. 2017;8:12. 28348722 10.4103/ijpvm.IJPVM_112_16 PMC5353762

[19] Raman TR, Devgan A, Sood SL, Gupta A, Ravichander B. LOW BIRTH WEIGHT BABIES: INCIDENCE AND RISK FACTORS. Med J Armed Forces India. 1998;54:191–5. 28775472 10.1016/S0377-1237(17)30539-7 PMC5531572

[20] Shah PS. Parity and low birth weight and preterm birth: a systematic review and meta-analyses. Acta Obstet Gynecol Scand. 2010;89:862–75. 20583931 10.3109/00016349.2010.486827

[21] Awoleke JO. Maternal risk factors for low birth weight babies in Lagos, Nigeria. Arch Gynecol Obstet. 2012;285:1–6. 21431841 10.1007/s00404-011-1885-y

[22] Nankabirwa JI, Gonahasa S, Katureebe A, Mutungi P, Nassali M, Kamya MR, Westercamp N. The Uganda housing modification study - association between housing characteristics and malaria burden in a moderate to high transmission setting in Uganda. Malar J. 2024;23:223. 39080697 10.1186/s12936-024-05051-5 PMC11290271

[23] Dike N, Onwujekwe O, Ojukwu J, Ikeme A, Uzochukwu B, Shu E. Influence of education and knowledge on perceptions and practices to control malaria in Southeast Nigeria. Soc Sci Med. 2006;63:103–6. 16448735 10.1016/j.socscimed.2005.11.061

[24] Kouamé RMA, Guglielmo F, Abo K, Ouattara AF, Chabi J, Sedda L, Donnelly MJ, Edi C. Education and Socio-economic status are key factors influencing use of insecticides and malaria knowledge in rural farmers in Southern Côte d'Ivoire. BMC Public Health. 2022;22:2443. 36577975 10.1186/s12889-022-14446-5 PMC9795670

